# Adjuvant instant preoperative renal artery embolization facilitates the radical nephrectomy and thrombectomy in locally advanced renal cancer with venous thrombus: a retrospective study of 54 cases

**DOI:** 10.1186/s12957-020-01985-7

**Published:** 2020-08-14

**Authors:** Guangxin Tang, Xiaoxu Chen, Jianwei Wang, Wei He, Zhihong Niu

**Affiliations:** 1 Shouguang People’s Hospital, Weifang, Shandong China; 2 Department of Pediatric Surgery, Jining First People’s Hospital, Jining, Shandong China; 3 Department of Urology, Shandong Provincial Hospital Affiliated to Shandong First Medical University, Jinan, Shandong China; 4 Department of Urology, Shandong Provincial ENT Hospital Affiliated to Shandong University, Jinan, Shandong Province China; 5 Department of Urology, Shandong Provincial Hospital, Cheeloo College of Medicine, Shandong University, Jinan, Shandong China

**Keywords:** Renal cell carcinoma, Embolization, Pre-operative, Nephrectomy, Tumor thrombus

## Abstract

**Background:**

The role of renal artery embolization (RAE) in the therapeutic armamentarium is always controversial. The present study aimed to assess the safety and the surgical outcomes of the instant renal artery embolization (I-RAE) prior to nephrectomy and thrombectomy in patients with locally advanced renal cell carcinoma (RCC) with venous thrombus.

**Methods:**

We performed a retrospective analysis of 54 patients treated with nephrectomy and thrombectomy between January 2012 and January 2019. Twenty-four patients were treated with I-RAE before surgery. Thirty patients received surgery alone (non-RAE group). The patient demographics, operation time, blood loss, transfusion requirements, complications, and other surgical parameters were analyzed between the two groups.

**Results:**

The mean tumor size in the I-RAE group was significantly larger than that in the non-RAE group (11.1 cm versus 7.9 cm; *p* = .001). The mean estimated blood loss was significantly lower in the I-RAE group compared to that in the non-RAE group (596 ml versus 827 ml; *p* = .015), and the patients in the non-RAE group were more likely to receive blood transfusion (red blood cell, RBC units, 4 U versus 6 U, *p* = .025; plasma volume, 200 ml versus 400 ml, *p* = .01). No differences were found in operative duration, ICU stay, perioperative complications, and length of postoperative hospitalization.

**Conclusions:**

Instant preoperative adjuvant renal artery embolization (I-RAE) is a safe technique. It facilitates nephrectomy and thrombectomy by reducing blood loss, transfusion requirements, and complications of delayed operations, providing urologists with a reliable option for treatment of locally advanced RCC with tumor thrombus.

## Background

Renal cell carcinoma (RCC) is the 6th most frequent cancer in men and 8th in women, accounting for 5% and 3% of all malignancies, respectively, with the incidence generally higher in developed countries. Up to 400,000 new cases were diagnosed, and ~ 175,000 deaths were recorded in 2018, worldwide [1]. Although most cases are diagnosed with small renal masses, a significant number of patients develop into the locally advanced stage and harbor metastasis. Up to 10% of cases are accompanied by intravascular invasion into the renal vein and inferior vena cava (IVC), forming tumor thrombus associated with poor prognosis [2]. In the past two decades, the surgical management of renal tumor has shifted significantly from an open approach to minimal-invasive surgeries. However, locally advanced renal tumors with venous thrombus are always surgical dilemmas for urologists.

Renal artery embolization (RAE) prior to surgery has been clinically practiced for over 40 years. In the 1970s, RAE was performed to control symptomatic hematuria and palliate metastatic RCC [3]. Later, the indication had extended to the management of angiomyolipomas, vascular malformations, traumatic renal hemorrhage, RCCs, and complications after renal surgeries [4]. Although many studies on RAE have been conducted, its role in the therapeutic armamentarium has always been controversial [5–7]. Therefore, we evaluated the surgical outcomes of instant renal artery embolization (I-RAE) in patients with locally advanced RCC with venous thrombus in our center.

## Methods

### Patients and study design

**Fig. 1:**
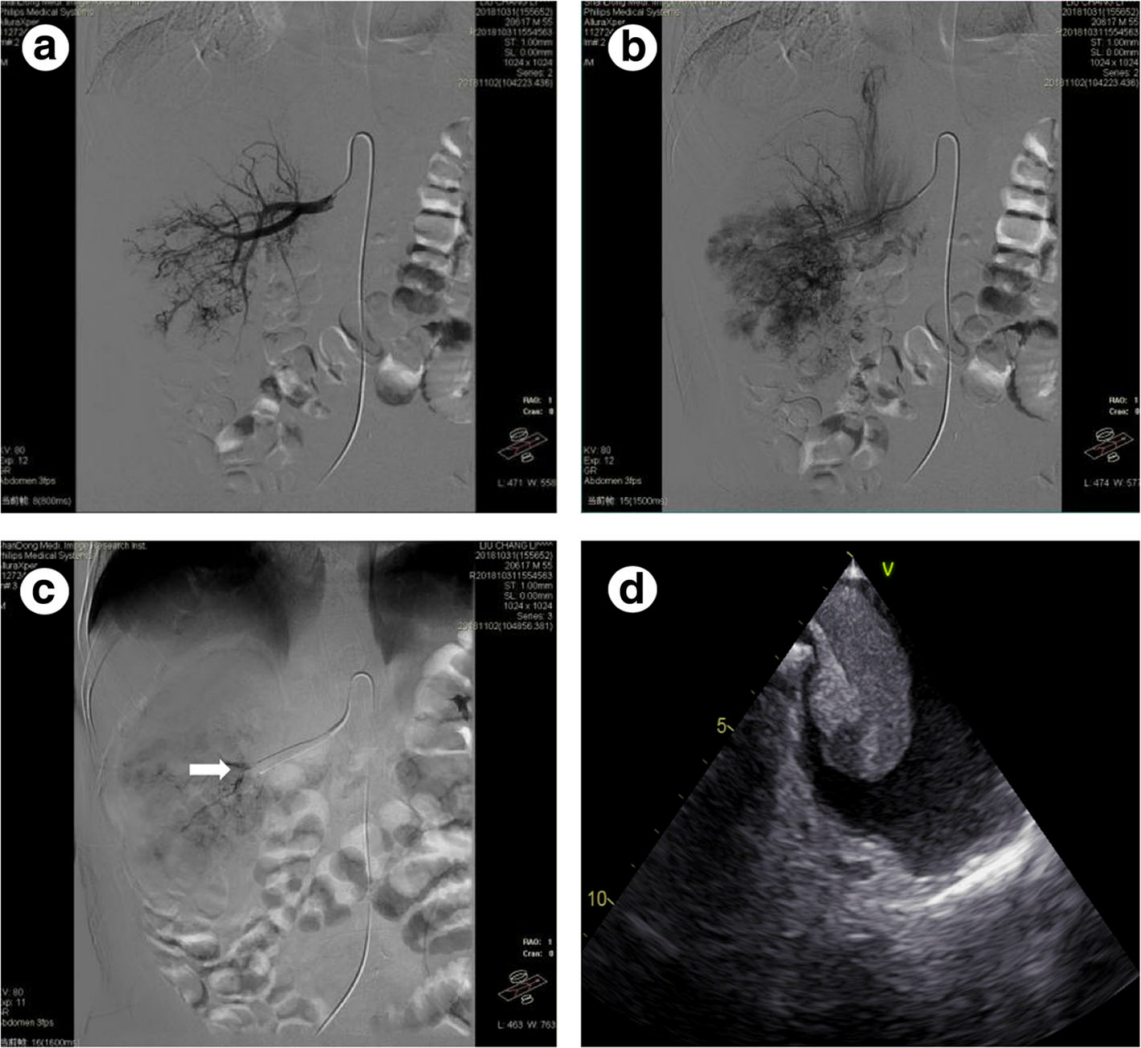
The renal arterial embolization of a 54-year-old renal cancer patient. **a**, **b** Renal arterial angiography demonstrating a right hypervascular renal cell carcinoma. **c** Performing the embolization of tumor vessels with gelatin sponge (white arrowhead). **d** The intraoperative trans-esophageal echocardiography showing the mobile thrombus mass extending across the tricuspid valve into the right ventricle

In this series, we retrospectively reviewed the patients with locally advanced renal cancer with venous thrombus who received radical nephrectomy and thrombectomy at our institution between January 2012 and January 2019. I-RAE was performed within 1 to 3 h prior to the operation. The right common femoral artery was prepared and draped in standard sterile fashion. After accessing the appropriate renal artery with the diagnostic catheter, angiography was obtained to visualize the renal artery system, RCC, and its supplying vessels (Fig. 1a, b). The vessels supplying the tumor were then selected using a micro-catheter and micro-wire. Angiography was again performed to confirm catheter location prior to embolization. A gelatin sponge was used as the embolic agent. All embolization was performed under careful fluoroscopy to prevent reflux into nontarget vessels. Technical success for embolization was defined as the stasis of all supplying arteries without further tumoral blush on postembolization angiography (Fig. 1c).

The surgery was performed within 3 h after embolization, to prevent the postinfarction syndrome and revascularization of the tumor. The strategy of surgery was as follows: (1) division of the renal hilar structures and ligation of the renal artery; (2) complete separation of the kidney and tumor; (3) mobilization of the IVC. The liver was mobilized to control the hepatic veins and IVC. The cardiopulmonary bypass was performed in the patients with suprahepatic and right atrial thrombus; and (4) completion of nephrectomy and thrombectomy or vena cavotomy with extraction of thrombus. We utilized cardiopulmonary bypass to facilitate the treatment of level IV tumor thrombus (Fig. 1d). The patient demographics, operation time, blood loss, transfusion requirements, complications, and other surgical parameters were analyzed between the two groups.

### Statistical analysis

Continuous variables were compared using Student’s *t* test for normally distributed data and the Mann-Whitney *U* test for nonnormally distributed data. Categorical variables were compared using the Pearson chi-square test. Statistical analysis was performed using SPSS version 24 (SPSS, Chicago, IL, USA); a *p* value < 0.05 was considered statistically significant.

## Results

Patient characteristics are summarized in Table 1. Overall, nephrectomy and thrombectomy were performed on 54 patients. The majority of patients were male (72.2%, 39/54). The mean age was 59.2 years, and the mean BMI was 23.3 kg/m^2^. The renal tumors were right-sided (*n* = 28, 51.9%) or left-sided (*n* = 26, 48.1%). Twenty-four patients were treated with I-RAE prior to surgery (I-RAE group, 24/54, 44.4%). Thirty patients were treated with surgery without embolization (N-RAE group, 30/54, 55.6%). The mean maximum diameter of the tumor in the I-RAE group was significantly larger than that in the non-RAE group (11.1 cm versus 7.9 cm, *p* = .001). Tumor thrombus was found at level 0 in 14 patients (6 versus 8), level I in 16 patients (9 versus 7), level II in 15 patients (6 versus 9), level III in 6 patients (2 versus 4), and level IV in 3 patients (1 versus 2), without statistical significance (*p* = .83). The clinical T-staging, NYHA, and ASA score systems are summarized in Table 1 without significances. There was no patient diagnosed with metastasis at the time of surgery.

**Table 1:** Patient characteristics

Characteristic	RAE (*n* = 24)	Non-RAE (*n* = 30)	*p* value
Age (years)	59.0 ± 11.8	59.3 ± 8.9	0.9
Sex (*n*)			0.15
Male	15 (62.5%)	24 (80%)	
Female	9 (37.5%)	6 (20%)	
BMI kg/m^2^	23.1 ± 2.8	23.4 ± 3.3	0.74
Affected kidney (*n*)			0.76
Right	13 (54.2%)	15 (50%)	
Left	11 (44.8%)	15 (50%)	
Tumor size (cm)	11.1 ± 3.5	7.9 ± 2.7	0.001*
Clinical T stage (*n*)			0.91
T3a	9 (37.5%)	10 (33.3%)	
T3b	13 (54.2%)	18 (60%)	
T3c	2 (8.3%)	2 (6.7%)	
Thrombus level (Mayo)			0.83
0	6 (25%)	8 (26.7%)	
I	9 (37.5)	7 (23.3%)	
II	6 (25%)	9 (30%)	
III	2 (8.3%)	4 (13.3%)	
IV	1 (4.2%)	2 (6.7%)	
NYHA classification			0.56
I	16 (66.7%)	16 (53.3%)	
II	7 (29.1%)	13 (43.3%)	
III	1 (4.2%)	1 (3.4%)	
ASA score			0.81
I	1 (4.2%)	1 (3.4%)	
II	8 (33.3%)	14 (46.6%)	
III	14 (58.3%)	14 (46.6%)	
IV	1 (4.2%)	1 (3.4%)	

*I-RAE* instant renal artery embolization, *BMI* body mass index

*p < 0.05

The operative outcomes are outlined in Table 2. In the I-RAE group, all patients underwent embolization within 3 h before surgery, and the mean interval was 128 min (57–172 min). Successful embolization was achieved in all cases. Three patients with mild flank pain were identified as having postinfarction syndrome (3/24, 12.5%), which was self-limited. There were no cases of embolic agent migration, adjacent organ injury, or other severe complications in the I-RAE group. Eighteen patients had laparoscopic surgery (8 in the I-RAE group, 10 in the non-RAE group), and the remaining patients received an open approach (16 in the I-RAE group, 20 in the non-RAE group). The mean operative time for the patients who had embolization was 219 min, while it was 233 min for the nonembolization group, without significant difference (*p* = .45). There was a significant difference between the two groups with regard to estimated blood loss (596 ml versus 827 ml; *p* = .015). Furthermore, the patients in the non-RAE group were more likely to receive a transfusion (62.5% [15/24] versus 90% [27/30]; *p* = .016). The transfused RBC units (median 4 U [range 2–6 U] versus median 6 U [range 2–8 U]; *p* = .025) and plasma volume (median 200 ml [range 200–600 ml] versus median 400 ml [range 200–800 ml]; *p* = .01) were significantly greater in the non-RAE group compared to the I-RAE group. No statistically significant differences were identified between the two groups with regard to the ICU stay (34 h versus 37 h; *p* = .58), surgical drainage (median 4 [range 2–16] versus median 3.5 [range 2–15]; *p* = .92), and postoperative hospitalization (median 7 [range 4–22] versus median 7 [range 4–15]; *p* = .67). The postoperative complications are outlined in Table 2 without significant differences between the two groups. Three cases were diagnosed with lower limb deep vein thrombosis. Three patients had acute kidney injury after nephrectomy. Four patients suffered a wound infection. Ileus was observed in two patients. All of them recovered after conservative treatment. No perioperative deaths occurred.

**Table 2:** Perioperative data

Variables	I-RAE	Non-RAE	*p* value
Surgery type			> 0.99
Open	16 (66.7%)	20 (66.7%)	
Laparoscopy	8 (33.3%)	10 (33.3%)	
Operative time (min)	219 ± 52	233 ± 75	0.45
EBL (ml)	596 ± 321	827 ± 347	0.015*
Transfusion rate	15/24 (62.5%)	27/30 (90%)	0.016*
RBC (U)			0.025*
Median, range	4 (2–6)	6 (2–8)	
Plasma (ml)			0.01*
Median, range	200 (200–600)	400 (200–800)	
ICU stay (h)	34 ± 10	37 ± 14	0.58
Postoperative hospitalization			0.67
Median, range	7 (4–22)	7 (4–15)	
Day to surgical drain removed			0.92
Median, range	4 (2–16)	3.5 (2–15)	
Interval (min)	128 ± 34	–	
Post-infarction syndromes			
Flank pain	3 (12.5%)	–	
Postoperative complications			
DVT	2 (8.3%)	1 (3.3%)	0.43
Acute kidney injury	2 (8.3%)	1(3.3%)	0.43
Wound infection	3 (12.5%)	1(3.3%)	0.21
Ileus	1 (4.2%)	1(3.3%)	0.87
Pathology			0.82
ccRCC	22 (91.7%)	28 (93.3%)	
Other	2 (8.3%)	2 (6.7%)	
Furhman grade			0.57
II	15 (62.5%)	17 (56.7%)	
III	7 (29.2%)	12 (40%)	
IV	2 (8.3%)	1 (3.3%)	

*I-RAE* instant renal artery embolization, *EBL* estimated blood loss, *Interval* interval between RAE and surgery, *DVT* deep vein thrombosis, *ccRCC* clear cell renal cell carcinoma

*p < 0.05

The pathology analysis of the tumors is summarized in Table 2. The histology type demonstrated a predominance of clear cell RCC in each group.

## Discussion

Vascular invasion is common in advanced renal cancer, which is associated with elevated morbidity and mortality. Surgically challenging radical nephrectomy and thrombectomy are considered as the standard treatment, showing prolonged survival [8]. RAE prior to surgery as adjuvant treatment in nephrectomy has been utilized for more than four decades. Craven et al. [9] reported that embolization minimized the oozing in the nephrectomy, controlled troublesome hematuria, and improved clinical status. Later, several studies showed that RAE reduced bleeding and surgical procedure time for nephrectomy, increasing the ease of dissection edematous tissue [10–12]. The embolization devascularized the tumor and allowed the renal vein to be ligated early, before control of the renal artery, without increasing the risk of tremendous hemorrhage from venous collaterals, which alleviated the nephrectomy in the cases with renal hilar structure invasion. This practice was also proposed to have immunological benefits, including augmentation of the natural killer cell and lymphoproliferative responses that are triggered by necrosis factor release, which caused the immune response [13–15].

Conversely, there were some conflicting data regarding the utility of adjuvant RAE prior to nephrectomy. A study [6] evaluated 227 patients with renal cancer who received embolization prior to nephrectomy matched with 607 patients treated with surgery alone. The investigators reported that there were no significant differences between the groups in complications and cancer-specific survival. However, the median follow-up was significantly lower in the surgical group compared to the embolization group. This study showed that blood transfusion requirements were significantly higher in the embolization group. The explanation for this could be the incomplete occlusion of the renal artery or obstruction of the IVC leading to hypertension of the bypass veins, which increased the hemorrhage of the venous collaterals around the renal capsule when mobilizing the kidney and tumor. In Subramanian’s study [7], 231 patients underwent radical nephrectomy and thrombectomy with 135 patients receiving preoperative embolization. It was reported that the patients in the embolization group had a longer median operative time (390 min versus 313 min) and received more blood transfusions compared to the control group (8 U versus 4 U). The authors pointed out that embolization was significantly associated with higher mortality (13% versus 3%). This series concluded that embolization did not show meaningful advantages. However, the embolization group was composed of higher tumor stages, IVC thrombus levels, ASA scores, and need for the utility of cardiopulmonary bypass. In addition, the patients treated with embolization almost had an association with hilar invasion and lymphadenopathy. These findings could explain the longer operative time and greater transfusion requirements in the embolization group.

In our study, we found that there was no significance in operative time between the two groups. One explanation could be that tumor size in the I-RAE group was larger than that in the non-RAE group, requiring more time for mobilizing and hemostasis. We also performed the RAE before laparoscopic surgery. Prophylactic embolization also had some merits in minimal-invasive surgery. Chopra and his colleagues [16] performed the preoperative embolization in 80% (20/24) of patients undergoing robot-assisted level II–III IVC thrombectomy. They concluded that RAE decompressed the venous collaterals, decreased blood loss, and enhanced robotic efficacy. Wang et al. [17] reported that the preoperative artery embolization could reduce intraoperative oozing, which was helpful for mobilizing the kidney and manipulating the vessels in robot-assisted IVC thrombectomy. Embolization was necessary and critical for left renal cancer, as the thrombectomy was performed in the left decubitus position. It was very difficult to expose the left renal artery. The embolization allowed the left renal vein to be disconnected well before the left renal artery can be robotically secured, intraoperatively.

Several types of materials were available for RAE, such as metallic coils, gelatin sponges, polyvinyl alcohol, embospheres, and *N*-butyl-2-cyanoacrylate (NBCA). We preferred the gelatin sponge, as it was cheapest. Its embolic effect could last for 2–3 weeks. In addition, it allowed surgical clamping and ligation during the nephrectomy without hindrance. The most common complaints after embolization were postinfarction syndrome, which is characterized by nausea, vomiting, fever, flank pain, malaise, hematuria, transient hypertension, and hyponatremia. The complications were self-limited and easily controlled with premedication and symptomatic treatment. RAE techniques have developed significantly over the past 20 years. Imaging capabilities have also improved dramatically. New embolic agents have allowed for more effective and precise embolization. Together, all of these decreased the complications caused by incomplete embolization or embolic material migration. Postinfarction syndrome always occurred 1–3 days after the embolization. For traditional embolization, the incidence of postinfarction syndrome ranged from 40 to 90% [18]. Kalman and Varenhorst [11] reported that the nephrectomy should be performed within 48 h. It became surgically difficult 3 days after the embolization, as there had been secondary collateral vessel formation. Minimizing the interval between RAE and surgery could decrease the incidence of postinfarction syndrome. Therefore, in this study, all the patients in the I-RAE group underwent surgery within 3 h of embolization, and we did not observe any major complications associated with RAE itself. The I-RAE had some advantages over the delayed surgery. First, the instant embolization alleviated the patients’ emotional strain and anxiety over waiting for several days. Second, as the nephrectomy was performed within 3 h, the occurrence of postinfarction syndrome was minimized. Some studies reported that if the nephrectomy was performed more than 4 days after the embolization, mortality may increase due to septic complications [19, 20]. Last, the instant approach reduced the time of hospitalization and cost compared to the delayed surgery.

To date, as there are no randomized, large-scale, prospective trials that compare the surgical outcomes of embolization and nonembolization, the European Association of Urology does not recommend the embolization as a routine procedure to manage RCC. However, in our study, the devascularization of tumor reduced intraoperative blood loss and transfusion, which facilitated the nephrectomy and thrombectomy in locally advanced RCC with large size tumor and hilar invasion. Prophylactic embolization could make some nonresectable renal masses resectable, providing urologists with a reliable option for locally advanced RCC. In addition, with the combination use of target drugs and immune-checkpoint inhibitors, the patients could benefit from the embolization-facilitated surgery.

## Conclusions

In conclusion, we reported our experience with the management of the I-RAE prior to nephrectomy in locally advanced RCC with venous thrombus. Our results have concluded that embolization is a safe technique, and it facilitates the surgery by reducing intraoperative blood loss, transfusion requirements, and complications of a delayed operation. Clearly, well-designed, large-scale, prospective randomized clinical trials are necessary to shed light on the pros and cons of RAE in nephrectomy.

## Supplementary information

**Additional file 1.** Detailed data of patients.

## Data Availability

The patient data will not be shared. All of the patient data was collected from the Shandong Provincial Hospital surgical and pathological databases. All patients provided written consent for the storage of their information in the hospital database only.

## Abbreviations

RCC: Renal cell carcinoma
RAE: Renal artery embolization
RBC: Red blood cell
IVC: Inferior vena cava
BMI: Body mass index
